# The Acute and Long-Term Benefits of the Oligoantigenic Diet for Children and Adolescents on the Three Symptom Subdomains of ADHD: Inattention, Hyperactivity, and Impulsivity

**DOI:** 10.3390/nu17111916

**Published:** 2025-06-03

**Authors:** Karolin Eder, Katja Schneider-Momm, Tanja Karola Puce, Maja Tobergte, Hans-Willi Clement, Reinhold Rauh, Eberhard Schulz, Monica Biscaldi, Christina Clement, Christian Fleischhaker

**Affiliations:** Department of Child and Adolescent Psychiatry, Psychotherapy, and Psychosomatics, Medical Center-University of Freiburg, Faculty of Medicine, University of Freiburg, D-79104 Freiburg, Germany; karolin.eder@uniklinik-freiburg.de (K.E.); katja.schneider-momm@uniklinik-freiburg.de (K.S.-M.); tanja.karola.puce@uniklinik-freiburg.de (T.K.P.); maja.tobergte@uniklinik-freiburg.de (M.T.); hans-willi.clement@uniklinik-freiburg.de (H.-W.C.); reinhold.rauh@uniklinik-freiburg.de (R.R.); profeberhardschulz@gmx.de (E.S.); monica.biscaldi-schaefer@uniklinik-freiburg.de (M.B.); christian.fleischhaker@uniklinik-freiburg.de (C.F.)

**Keywords:** ADHD, diet, children, nutrition, food intolerance

## Abstract

**Background:** Based on the multitude of findings, nutrition is becoming increasingly important in the treatment of attention deficit/hyperactivity disorder (ADHD) in children. One promising approach is the so-called oligoantigenic diet (OD). This intervention involves avoiding certain foods that often trigger intolerances and allergies. Previous studies have shown that around 60% of patients experienced a significant reduction in ADHD symptoms after completing such a diet. The aim of the present study was to further confirm the efficacy of the OD within an analysis focusing on the symptom of impulsivity. **Materials and Methods:** In the present study, the Parent Rating of the Diagnostic System of Mental Disorders in Children and Adolescents (DISYPS-II FBB-ADHD) questionnaire was used to measure the severity of ADHD symptoms. Of 34 children and adolescents (between 7 and 18 years of age) screened and included in this study, 31 participants completed the 4-week OD diet. **Results:** The corresponding post-diet analysis showed significant short-term improvements for the DISYPS-II FBB-ADHD total score, compared to the start of the diet. This pattern of results also applied to the respective subscales of the DISYPS-II FBB-ADHD questionnaire. A follow-up evaluation conducted 3.5 years after the intervention with 21 participants suggested that the improvements in ADHD symptoms were maintained over time. Specifically, 66.7% of the participants continued to meet the responder criterion, with particularly notable and lasting reductions in impulsivity. **Discussion:** These results suggest that the beneficial effects of the oligoantigenic diet followed by identifying and avoiding individual intolerant foods may persist long term, and participants’ dietary habits may have also evolved over the years. **Conclusion:** The oligoantigenic diet may have long-term therapeutic potential for reducing ADHD symptoms, especially impulsivity, in children and adolescents.

## 1. Introduction

Attention deficit/hyperactivity disorder (ADHD) has undergone sustained development and continuous refinement of its definition and understanding over the last five decades. It is classified as a neurodevelopmental disorder marked by persistent patterns of inattention, hyperactivity, and impulsivity that interfere with daily functioning. Impulsivity, in this context, refers to rapid, unplanned reactions to stimuli with limited regard for consequences, such as interrupting others or difficulty delaying responses [1]. The worldwide prevalence varies greatly (depending on geographical, cultural, and diagnostic factors) between 5 and 10% for children and 4% for adults. Despite the multitude of findings, the appropriate treatment of ADHD remains a very complex issue. Pharmacotherapy (and psychostimulants in particular) is the most common treatment for ADHD in children, adolescents, and adults with a response rate of around 70% in children aged five or older (Krinzinger et al., 2019) [2].

However, pharmacological therapy can cause negative side effects and long-term consequences, such as gastrointestinal diseases and sleep disturbances [3]. These effects vary between patients, due to a multifactorial interaction of psychological aspects, genetics, and neurological structures [4]. As a result, non-pharmacological approaches are increasingly being researched and used, either as a supplement to medication or as a substitute [5,6,7]. Interestingly, the previous research has suggested that external factors can also increase or decrease ADHD symptoms in patients, which results in further potential treatment methods. For example, the significant influence of food intolerances on behavioral disorders was already introduced 100 years ago by Shannon, 1922 [3], and later supported by Crook et al., 1961 [8]. According to this hypothesis of food intolerance, avoiding certain foods (such as milk and chocolate) as part of a diet could reduce the corresponding symptoms of behavioral disorders. Since then, numerous findings have demonstrated the significant influence of nutrition, particularly regarding ADHD symptoms. For example, children with ADHD show a significantly higher prevalence of lactose intolerance [9]. Endreffy et al. [10] provided evidence that carbohydrate (oligosaccharide) metabolism differs in children with ADHD (compared to healthy children). This raises the question of whether dietary control influences any of the metabolic disorders observed in ADHD. In addition, several studies showed an association between ADHD symptoms and celiac disease. However, a gluten-free diet led to a significant reduction in the corresponding symptoms [11]. These results emphasize the potential of non-drug methods in the treatment of ADHD. Corresponding approaches have been intensively researched in recent years. For example, the use of gut microbiomes led in part to a significant reduction in ADHD symptoms [12,13] but also revealed limitations in relation to genetic factors.

A more promising approach is the oligoantigenic diet (OD), introduced by Egger et al., 1985 [14]. This dietary approach avoids foods that are associated with intolerances or allergies. In the corresponding study, the participants ate hypoallergenic foods following previous studies on migraine in children [15,16]. Apart from vitamin supplementation and calcium-rich water, the diet contained vegetables, two fruits, two sources of carbohydrates, and two types of meat. A total of 82% of all participants showed a significant reduction in ADHD symptoms. In addition, 29% of all participants showed normal behavior at the end of this study. Several recent studies have demonstrated this significant impact of the OD [17,18,19]. Furthermore, Dölp et al. [17] found no difference in reliability between the unblinded and blinded assessments of the ADHD Rating Scale IV (ARS) for assessing the impact of the OD.

In this paper, we evaluate the long-term effects of a 4-week oligoantigenic diet on ADHD symptoms in children with a special focus on impulsivity. Patients who responded to a four-week oligoantigenic diet with a severe reduction in ADHD symptoms identified their individual food intolerances during a subsequent food reintroduction phase. On the basis of these findings, it was recommended to avoid these foods causing intolerances for at least one year. The effect of the dietary intervention is based on four weeks of oligoantigenic diet followed by a minimum of one year avoidance of individually intolerant foods. Symptom severity was captured with the DISYPS-II FBB-ADHD, a validated German parent-rating scale whose psychometric quality and administration are described in the Methods section. Previous research by Walz et al. [18] demonstrated that such an intervention can lead to a significant and sustained reduction in ADHD symptoms with improvements persisting over a 3.5-year follow-up. Given the high importance of long-term outcomes [20], our study aims to further explore these findings by examining the impact and durability of the diet-induced symptom reduction.

## 2. Materials and Methods

The Ethics Committee of the University of Freiburg (application number 111/14, date of approval 13 October 2014) approved this study in accordance with the World Medical Association’s Declaration of Helsinki.

### 2.1. Participants

Recruitment took place at the inpatient and outpatient units of the Department of Child and Adolescent Psychiatry, Psychotherapy, and Psychosomatics of the Medical Center (University of Freiburg, Freiburg im Breisgau, Germany). This study included children and adolescents aged 7 to 18 who were attending at least the 2nd grade of a general education school and had a confirmed ADHD diagnosis according to both the DSM-IV and ICD-10 criteria. Participation required agreement with the study procedure and informed consent from both the participants and their parents. The exclusion criteria included severe concomitant diseases or neurological or organic comorbidities not amenable to dietary interventions, lack of compliance from parents and/or children, insufficient reading or writing skills of legal guardians and/or children, concurrent drug therapy for ADHD, participation in other studies, or adherence to a special diet (e.g., vegetarian, vegan).

Table 1 shows the characteristics of the participants after the OD intervention and the FU. A total of 34 children took part in this study between 2014 and 2023. After the completion of the OD, 31 participants with a mean age of *M* = 10.50 (*SD* = 2.28) years were included in this study. In the follow-up analysis, data from 21 participants were available, with a mean age of *M* = 14.10 (*SD* = 2.09) years.

### 2.2. Procedure

**Figure 1:**
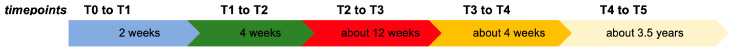
Citation.

Figure 1 below illustrates the respective phases of the study, which will be explained in more detail.

#### 2.2.1. Pre-Diet Phase (T0–T1)

Participants were enrolled between 2014 and 2017, with additional recruits in 2021. At the baseline (T0, see Figure 1) assessment, the ADHD diagnoses were confirmed using the Kiddie-SADS-PL interview [21]. Comprehensive medical histories, including symptoms and allergies, were collected, followed by psychiatric and medical examinations. Following the baseline assessment, a two-week pre-diet phase (T0–T1) commenced. During this period, participants maintained their usual dietary habits. Parents precisely recorded all food and beverage intake in detailed food diaries. This phase also served to prepare participants for the subsequent diet phase.

#### 2.2.2. Diet Phase (T1–T2)

During the four-week diet phase (T1–T2), participants adhered to a restricted diet of oligoantigenic foods with low allergenic potential, according to the protocols of Egger [14] and Pelsser et al. [22]. Excluded from the diet were pig and cow meat, wheat, soy, and corn products, while lamb, turkey, potatoes, rice, and various vegetables were permitted. Parents received comprehensive dietary instructions, including lists of acceptable foods, grocery lists, and recipes. Family members were encouraged to follow the diet to support adherence. Participants attending full-time school were required to consume home-prepared meals. A nutritionist supervised the diet to ensure it met all essential nutritional requirements and provided guidance on supplementation when necessary.

#### 2.2.3. Reintroduction Phase (T2–T4)

Participants whose ARS value decreased by more than 40% between T1 and T2 were classified as responders, according to Pelsser et al. [23]. Non-responders could finish this study and receive the usual treatment. Responders began the reintroduction phase (T2–T4) to gradually return to their usual diet, testing new foods every three to four days in this order: milk products, favorite foods, eggs, grain products, fish, meat, vegetables, fruits, and nuts. If behavioral changes or symptoms such as abdominal pain, headaches, or allergic reactions occurred, the psychiatrist and dietitian were notified, and the triggering food was avoided. Reactions could occur from ten minutes to several days after ingestion [14]. Only the tolerated foods were consumed for three days after each test. Approximately six weeks after the beginning of the reintroduction phase, a control appointment (T3) was conducted. After testing all common nutrients, the final study appointment (T4) took place, where personalized dietary recommendations were provided. Children were advised to avoid problematic foods for one year, after which these foods could be retested.

#### 2.2.4. Follow-Up (T5)

The follow-up examination occurred 2.50 to 5.37 years after the diet started (*M* = 3.33 years, *SD* = 1.15). Both responders and non-responders were re-interviewed to evaluate their benefits from the diet.

During this period, adherence to the original oligoantigenic diet was required for responders, and the individual intolerant foods should be avoided. But it has to be reported that short-term dietary interruptions occurred, which can be assumed as not causing effects on long-term follow-ups; the non-responders reported an eating pattern as usual. Accordingly, the long-term outcomes were assessed using standardized interviews. This approach allowed us to capture the sustainability of the initial treatment effects while accommodating individual modifications in “dietary behavior over time”.

#### 2.2.5. Outcome Measures

The primary outcome was assessed using the ADHD Rating Scale IV (ARS), consisting of 18 items divided equally between the inattention and hyperactivity/impulsivity subscales. Each question is rated as “Never or rarely” (0), “Sometimes” (1), “Often” (2), or “Very often” (3). The ARS was conducted at each appointment by the study clinician, who interviewed the parents in the presence of the child. A symptom improvement of more than 40% between the two appointments, T1 (before the diet) and T2 (after the diet), was defined as a response.

Three questionnaires were used as secondary outcome measures: the Child Behavior Checklist *4-18* (CBCL/4-18), the Inventory of Life Quality in Children and Adolescents (ILC), and the DISYPS-II FBB-ADHD [17,18].

For this study, the DISYPS-II FBB-ADHD was used for a more detailed assessment of ADHD symptoms. The assessment form is suitable both for confirming the diagnosis and for monitoring the progress of the ADHD symptoms. In addition to the version as an external assessment form (FBB), it is also available as a diagnostic checklist for clinical assessors (DCL) and as a self-assessment form (SBB) for children and adolescents from the age of 11.0 to 17.11 years [24,25]. The DISYPS-II FBB-ADHD questionnaire consists of 35 items designed for diagnostic confirmation and symptom monitoring. The first part contains 20 items, while the second part includes 15 items organized into three blocks: A (distress), B (symptom occurrence), and C (competence). The instrument comprises a total scale and three subscales: inattention, hyperactivity, and impulsivity, with hyperactivity and impulsivity treated as separate subscales. Each item is rated on a four-point rating scale from 0 (not at all) to 3 (very much). In practice, the DISYPS-II FBB-ADHD has been used in numerous studies, particularly those involving medication therapy. However, it has not been used in the context of dietary interventions such as the oligoantigenic diet.

Regarding the reliability and validity of the DISYPS-II FBB-ADHD, a study by Görtz-Dorten and Döpfner [25] highlighted its distinctive three-factor structure (Inattention, Hyperactivity, Impulsivity), which reflects the core symptoms of ADHD. It is the only ADHD-specific rating scale to also assess competencies (section K), potentially making it more sensitive to therapy-related changes. The reliability of the subscales and overall ADHD score ranged from satisfactory to very good, aligning with international reliability values.

### 2.3. Statistical Analysis

Analysis of DISYPS-II FBB-ADHD consisted of two different parts depending on different time points. Firstly, the immediate effect of the OD was analyzed by comparing the DISYPS-II FBB-ADHD scores at the pre-diet phase and the post-diet phase (post-diet analysis). In addition, the long-term effect of the OD was analyzed by comparing the DISYPS-II FBB-ADHD scores between pre-diet, post-diet, and follow-up timepoints (follow-up analysis). For both analyses, only participants with complete data sets were accordingly included. A repeated-measures ANOVA (rmANOVA) was conducted to investigate possible differences between the corresponding time points. Post hoc tests were performed using a Bonferroni correction to compare the different time points. All statistical analyses were performed with R Statistical Software (v4.1.2; R Core Team, 2021 [26]).

## 3. Results

### 3.1. Participants

Table 2 shows an overview of the study participants. After the screening, 34 participants started the diet, and 31 participants completed the diet. At the follow-up, data were available for 21 participants. Of these 21 participants, 14 (=66.7%) participants fulfilled the responder criterion, while 7 (=33.3%) participants did not. Table 3 provides an overview of the medication used by responders and non-responders.

### 3.2. Post-Diet Analysis

The data from 31 participants were available for analyses using repeated measures ANOVA (rmANOVA) on the DISYPS-II FBB-ADHD. The scores after completion of the OD (post-diet) were compared with the scores before the start of the diet (pre-diet). In addition to the total score, the subscale scores for impulsivity, inattention, hyperactivity, and competence were also calculated. The descriptive statistics and the rmANOVA results are displayed in Table 4.

### 3.3. Total Score

The DISYPS-II FBB-ADHD total score decreased between the pre-diet timepoint (with *M* = 1.72 and *SD* = 0.52) and the post-diet timepoint (with *M* = 1.03 and *SD* = 0.62). The rmANOVA revealed a significant effect of time (*F*(1, 30) = 51.37, *p* < 0.001, Cohen’s *d* = 1.20).

#### 3.3.1. Impulsivity

The DISYPS-II FBB-ADHD impulsivity score also decreased between the pre-diet timepoint (with *M* = 1.59 and *SD* = 0.80) and the post-diet timepoint (with *M* = 0.93 and *SD* = 0.90). Again, the rmANOVA revealed a significant effect of time (*F*(1, 30) = 20.78, *p* < 0.001, Cohen’s *d* = 0.78).

#### 3.3.2. Inattention

Furthermore, the DISYPS-II FBB-ADHD inattention score also decreased between the pre-diet timepoint (with *M* = 2.06 and *SD* = 0.54) and the post-diet timepoint (with *M* = 1.32 and *SD* = 0.60). Again, the rmANOVA revealed a significant effect of time (*F*(1, 30) = 54.86, *p* < 0.001, Cohen’s *d* = 1.32).

#### 3.3.3. Hyperactivity

In addition to the previous results, the DISYPS-II FBB-ADHD hyperactivity score also decreased between the pre-diet timepoint (with *M* = 1.36 and *SD* = 0.75) and the post-diet timepoint (with *M* = 0.73 and *SD* = 0.69). Again, the rmANOVA revealed a significant effect of time (*F*(1, 30) = 28.81, *p* < 0.001, Cohen’s *d* = 0.87).

#### 3.3.4. Competence

This pattern of results was also observed for the DISYPS-II FBB-ADHD competence score, which increased between the pre-diet timepoint (with *M* = 0.69 and *SD* = 0.47) and the post-diet timepoint (with *M* = 0.94 and *SD* = 0.59). Again, the rmANOVA revealed a significant effect of time (*F*(2, 48) = 11.02, *p* < 0.01, Cohen’s *d* = −0.45).

### 3.4. Post-Diet Analysis: Changes in Statement Blocks A and B

The DISYPS-II FBB-ADHD contains items on various statements in German, divided into Block A and Block B. Block A refers to the overall burden and impairments, while Block B examines the degree of generalization of the symptoms.

In Block A, statistically significant changes were observed in item A1 (“The behavioural problems are very stressful overall”; *p* = 0.02, Cohen’s *d* = 0.89) and item A3 (“The behavioural problems affect the relationship with adults”; *p* < 0.01, Cohen’s *d* = 1.2). In contrast, no statistical significance was found for item A2 (“The behavioural problems impair academic performance”) or item A4 (“The behavioural problems impair relationships with children or adolescents”).

Block B assesses the occurrence of symptoms across different environments. Interestingly, significant changes were observed in item B1 (“The behavioural problems occur in the family”; *p* < 0.01, *d* = 1.22) and item B3 (“The behavioural problems occur outside the family and kindergarten/school”; *p* = 0.04). However, item B2 (“The behavioural problems occur in kindergarten or at school”) did not show statistical significance.

### 3.5. Follow-Up Analysis

In total, the results of 21 participants were used for the analyses of the DISYPS-II FBB-ADHD results between the pre-diet, post-diet, and follow-up timepoints. As a smaller sample was used in this analysis, the difference between the pre-diet and post-diet was also reconsidered. Again, the scores for the impulsivity, inattention, hyperactivity, and competence subscales were also calculated, in addition to the total score. Descriptive statistics and the rmANOVA results are displayed in Table 5.

### 3.6. Total Score

**Figure 2:**
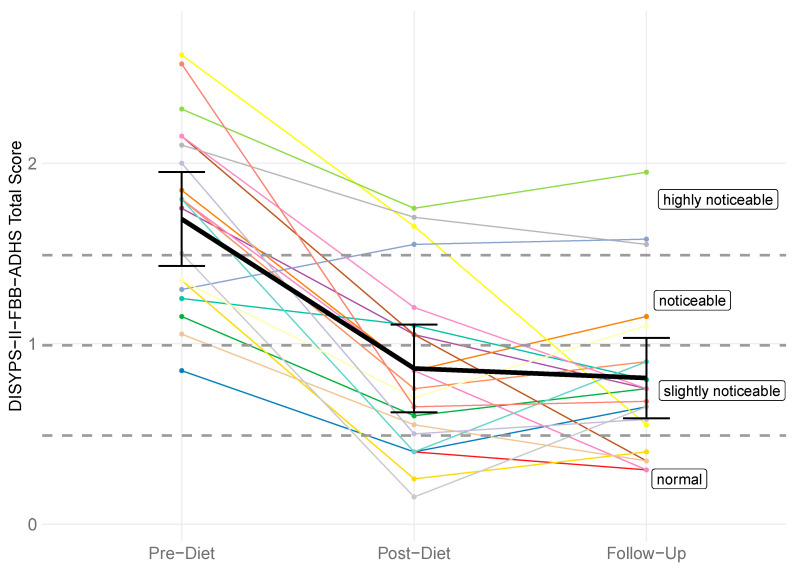
DISYPS-II FBB-ADHD total scores for all participants (*n* = 21) across three time points: pre-diet, post-diet, and follow-up. Each line represents one participant. A reduction in scores indicates a decrease in symptom severity.

There was a significant difference in the DISYPS-II FBB-ADHD total score between the pre-diet timepoint (*M* = 1.69, *SD* = 0.52) and the follow-up timepoint (*M* = 0.81, *SD* = 0.45). The rmANOVA revealed a significant effect of time (*F*(2, 40) = 39.64, *p* < 0.001, *η*^2^*_p_* = 0.42). Subsequent post hoc tests also revealed a significant lower total score at the post-diet timepoint (*M* = 0.86, *SD* = 0.49) compared to the pre-diet timepoint, with *p* < 0.001. However, there was no significant change between the post-diet and the follow-up timepoint (*p* = 0.999). Figure 2 shows the individual DISYPS-II FBB-ADHD total scores for all participants.

#### 3.6.1. Impulsivity

**Figure 3:**
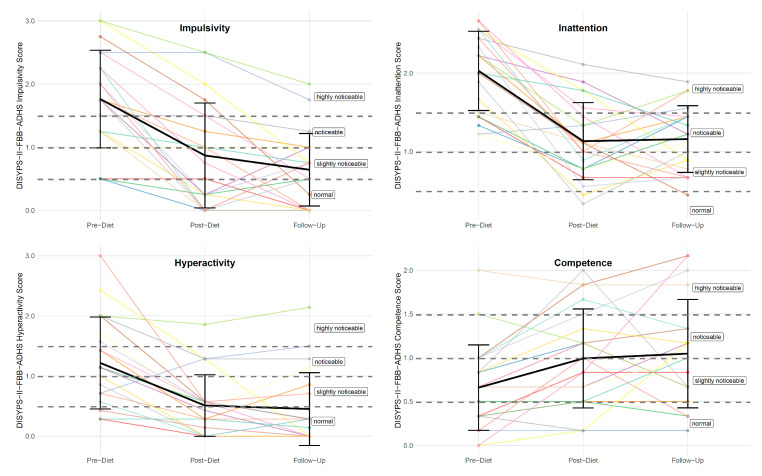
Individual DISYPS-II FBB-ADHD subscale scores (*n* = 21) across the three time points: pre-diet, post-diet, and follow-up. Each line represents one participant.

There was also a significant difference in the DISYPS-II FBB-ADHD impulsivity score between the pre-diet timepoint (*M* = 1.76, *SD* = 0.77) and the follow-up timepoint (*M* = 0.64, *SD* = 0.57). The rmANOVA revealed a significant effect of time (*F*(2, 40) = 29.90, *p* < 0.001, *η*^2^*_p_* = 0.31). Subsequent post hoc tests also revealed a significant lower total impulsivity at the post-diet timepoint (with *M* = 0.87, *SD* = 0.83) compared to the pre-diet timepoint, with *p* < 0.001. Interestingly, the impulsivity score further decreased after the completion of the diet, with *M* = 0.64 (*SD* = 0.57) at the follow-up timepoint. However, this reduction did not reach statistical significance (*p* = 0.405). Figure 3 shows the individual DISYPS-II FBB-ADHD impulsivity scores for all participants.

#### 3.6.2. Inattention

Furthermore, there was a significant difference in the DISYPS-II FBB-ADHD inattention score between the pre-diet timepoint (*M* = 2.03, *SD* = 0.50) and the follow-up timepoint (*M* = 1.16, *SD* = 0.43). The rmANOVA revealed a significant effect of time (*F*(2, 40) = 34.72, *p* < 0.001, *η*^2^*_p_* = 0.45). Subsequent post hoc tests also revealed a significant lower inattention score at the post-diet timepoint (*M* = 1.13, *SD* = 0.49) compared to the pre-diet timepoint, with *p* < 0.001. However, there was no significant change between the post-diet and the follow-up timepoint (*p* = 0.999). See Figure 3.

#### 3.6.3. Hyperactivity

In addition, there was a significant difference in the DISYPS-II FBB-ADHD hyperactivity score between the pre-diet timepoint (*M* = 1.22, *SD* = 0.76) and the follow-up timepoint (*M* = 0.45, *SD* = 0.60). The rmANOVA revealed a significant effect of time (*F*(2, 40) = 20.06, *p* < 0.001, *η*^2^*_p_* = 0.24). Subsequent post hoc tests also revealed a significant lower hyperactivity score at the post-diet timepoint (*M* = 0.51, *SD* = 0.51) compared to the pre-diet timepoint, with *p* < 0.001. However, there was no significant change between the post-diet and the follow-up timepoint (*p* = 0.999) (see Figure 3).

The “omnibus” test (Table 6) was used to check whether there is a difference in the course over the three time points (pre-diet, post-diet, follow-up) of the three symptom categories “inattention”, “hyperactivity”, and “impulsivity”.

Two approaches were used for the statistical evaluation, a univariate analysis, which offers a higher test strength (power), but requires stronger assumptions about the distribution structure of the data and a multivariate analysis, which has fewer distribution requirements, but is associated with a lower test strength.

Both approaches show significant main effects for the factors of “time” and “symptom category”. The main effect of time is due to the fact that the mean values drop significantly from pre- to post-diet. The main effect of symptom category shows that across all time points, the ratings for inattention are systematically higher than those for impulsivity, which in turn are consistently higher than the ratings for hyperactivity. With regard to the interaction, the question arises as to whether the three symptom categories develop differently over time. No significant interaction effect was found in either analysis approach. However, the univariate approach narrowly misses the significance level (*F*(4, 80) = 2.20, *p* = 0.076). The graphic representation shows that the impulsivity values tend to continue to decrease from post-diet to follow-up.

The univariate analysis indicates that the interaction is close to the significance limit. In this case, it would be appropriate to argue that the sample size may not be sufficient to detect smaller effects and that future studies with larger numbers of cases should examine whether the course of impulsivity actually differs from the other symptom categories. This question could be of particular interest for psychiatric disorders in which impulsivity plays a central role. With the focus on the multivariate analysis, the emerging interaction effect could be omitted from highlighting and instead the results of the multivariate analysis could be presented, which more clearly reflect the lack of significance of the interaction.

#### 3.6.4. Competence

This pattern of results was also observed for the DISYPS-II FBB-ADHD competence score. There was a significant difference in the DISYPS-II FBB-ADHD competence score between the pre-diet timepoint (*M* = 0.66, *SD* = 0.49) and the follow-up timepoint (*M* = 1.05, *SD* = 0.62). The rmANOVA revealed a significant effect of time (*F*(2, 40) = 6.23, *p* < 0.01, *η*^2^*_p_* = 0.09). Subsequent post hoc tests also revealed a significant higher competence score at the post-diet timepoint (*M* = 0.99, *SD* = 0.57) compared to the pre-diet timepoint, with *p* < 0.01. However, there was no significant change between the post-diet and the follow-up timepoint (*p* = 0.999) (see Figure 3).

### 3.7. Responder and Non-Responder Results

**Figure 4:**
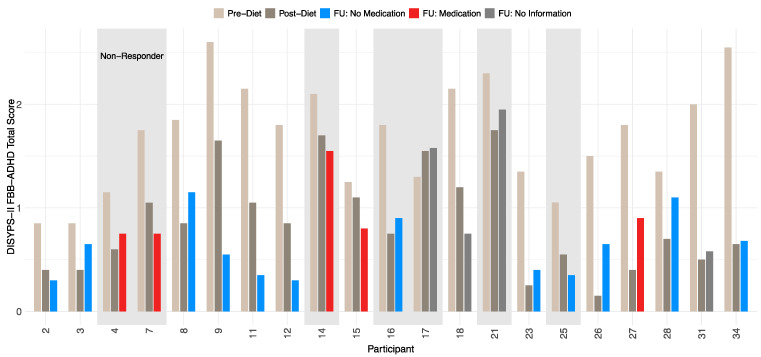
Time course of DISYPS-II FBB-ADHD **total scores** for responders and non-responders (*n* = 21) according to ARS-rating depending on medication use. Scores are shown at pre-diet, post-diet, and follow-up. Missing medication data at follow-up for participants 17, 18, 21, and 31. Lower scores indicate fewer ADHD symptoms.

**Figure 5:**
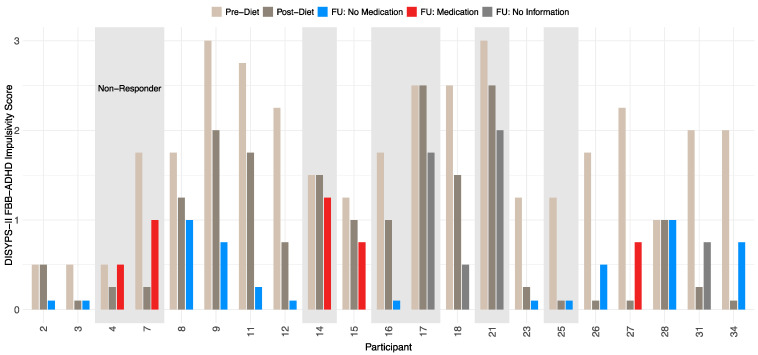
Time course of DISYPS-II FBB-ADHD **impulsivity scores** for responders and non-responders (*n* = 21) according to ARS-rating depending on medication use. The short bars represent an impulsivity score of 0. No information on medication was available at the FU for participants 17, 18, 21, and 31.

In this study, responders were defined as participants who showed a 40% (or greater) reduction in the ARS total score between the pre-diet and the post-diet timepoints. At the follow-up, 14 participants were responders, resulting in a responder rate of 66.7%. A total of seven participants were considered as non-responders. All participants showed a significant reduction in symptoms after completing the diet, which remained stable even after a period of 3.5 years. A more detailed development of the DISYPS-II FBB-ADHD total score over time can be found in Table 7. The mean DISYPS-II FBB-ADHD total score decreased in the responders from the pre-diet to the follow-up timepoints. Interestingly, the medication had no significant influence on this decrease. However, due to the small sample in this analysis, only two participants were considered as a responder with medication use, which can fully explain this finding. In addition, an improvement in the DISYPS-II FBB-ADHD total score was also observed in the non-responders. Nevertheless, only seven participants were considered to be non-responders. The statistical significance of this development is therefore limited. In Figure 4, the individual DISYPS-II FBB-ADHD total scores are visualized for each participant. In addition, the individual DISYPS-II FBB-ADHD impulsivity scores are displayed in Figure 5.

## 4. Discussion

The aim of the present study was to evaluate the long-term effects of an oligoantigenic diet on ADHD symptoms in children. The DISYPS-II FBB-ADHD questionnaire was used to measure the severity of ADHD symptoms. Our results provide further evidence for the significant reduction in the corresponding symptomatology in children who took part in a four-week oligoantigenic diet. This effect is therefore clearly not dependent on the questionnaires used. Furthermore, this improvement has not deteriorated in a period of 3.5 years, which indicates the desired long-term effects. In order to provide a concise overview of our most important findings, we summarize the key outcomes as follows:

- The intervention produced a statistically significant reduction in ADHD symptoms across all core domains, including inattention, hyperactivity, and impulsivity.
- Sustained clinical benefits at the 3.5-year follow-up, suggesting long-term efficacy.
- A responder rate of 66.7%, consistent with previous research, supporting the robustness of the oligoantigenic diet.

These key findings are discussed in further detail below, in relation to the current literature, the potential mechanisms of action, and the limitations of our study.

Following the work of Yorgidis et al. [19], we found a similar pattern of results with significant improvements on all subscales of the DISYPS-II. Furthermore, we observed a responder rate of 66.7%, which is almost identical to the responder rate of 64% reported in the paper by Walz et al. [18]. Both result patterns thus support the work of Egger [14] and Pelsser [22], who reported responder rates of 60% and the previous findings in general [17,23,27,28]. By contrast, Schmidt et al. [29] reported a markedly lower responder rate of 24%, possibly due to a substantially shorter intervention duration of only nine days. The stronger long-term effects observed in our study may result from the four-week intervention, consistent adherence, and extended follow-up, which together captured improvements overlooked in shorter studies.

In the past, the effectiveness of elimination diets (such as the OD) has often been analyzed in direct comparison to healthy diets [30]. While an elimination diet aims to identify specific dietary triggers that could exacerbate ADHD symptoms, a healthy diet emphasizes nutrient-rich foods (without specifically avoiding possible triggers) to ensure a balanced diet. In two recent studies, Huberts-Bosch et al. [31,32] emphasized the limited benefits of elimination diets, both in the short and long term. In this context, the authors criticized the work of Walz et al. [18] for overestimating the percentage of responders. Furthermore, Huberts-Bosch et al. drew the conclusion that the initial improvements caused by an elimination diet diminish over time. However, the design of this study itself leads to a decisive weakness in this conclusion: short-term benefits were analyzed after a period of five weeks, while long-term benefits were analyzed after a period of more than one year. In contrast, the present study used a follow-up period of 3.5 years to analyze the long-term improvements of an OD (following Walz et al. [18]). The results are therefore only comparable to a limited extent. Differences in dietary protocols across studies might help explain variations in long-term effects, and future research should further investigate how varying degrees of food exclusion impact the therapeutic outcomes.

In a study by Meßler et al. [33], high-intensity interval training (HIIT) combined with multimodal therapy led to moderate improvements in social competencies (Cohen’s *d* = 0.59), but 35.7% of participants in the HIIT group also received concurrent medication. In contrast, our study excluded pharmacological treatment yet achieved stronger effect sizes (Cohen’s *d* = 1.20), suggesting that dietary intervention alone can produce robust effects.

Further comparisons support the clinical relevance of OD. Gevensleben et al. [34] reported effect sizes of 0.6 (total), 0.57 (inattention), and 0.45 (hyperactivity/impulsivity) for neurofeedback training (*n* = 35), while our study showed 1.20, 1.32, and 0.87 (summarized for comparison), respectively. Sinzig et al. [35] examined methylphenidate treatment using the DISYPS-II parent rating and found effect sizes of 1.55 (total), 1.68 (inattention), 1.50 (hyperactivity), and 0.99 (impulsivity). Although pharmacological interventions tend to yield the strongest results, OD demonstrates clinically meaningful effects without medication and may serve as a viable complementary or alternative approach. In clinical cases with severe impulsivity, antipsychotic medication is used for treatment [36,37,38,39,40,41]. As demonstrated, the OD might be an alternative treatment in such cases.

A central line of criticism concerns the potential impact of restrictive diets on the intestinal microbiome. Recent findings have emphasized the importance of microbial diversity for immune function, tissue repair, and neurological processes. Di Sabatino et al. [42] linked low biodiversity to insufficient anti-inflammatory responses, which may impair neurological processes, including in the brain. Dysbiosis has also been associated with several chronic and neurodevelopmental conditions, including ADHD, inflammatory bowel disease, obesity, cardiovascular disease, and autoimmune disorders [42,43,44,45,46,47,48]. Although concerns have been raised that restrictive diets may reduce microbial diversity, the OD is designed as a temporary diagnostic tool, followed by the controlled reintroduction of tolerated foods. It therefore does not inherently contradict the objective of promoting microbiota resilience.

The experimental evidence supports the relevance of gut–brain interactions in behavioral disorders. In an animal study, Leo et al. [49] demonstrated that combining pre- and post-biotics improved behavioral outcomes in mouse models of autism and depression, likely through modulation of the gut microbiota. These results reinforce the broader understanding that targeted nutritional strategies can influence mental health. While the OD operates through a different mechanism—namely the identification of food-related triggers—both approaches highlight the growing importance of personalized dietary interventions in neurodevelopmental conditions.

Despite its demonstrated clinical efficacy, the precise mechanisms underlying the effects of OD remain unclear. A direct causal link between food intolerances and ADHD symptoms has not yet been conclusively established, but studies have repeatedly shown associations between ADHD and allergic conditions [44,45,46], as well as altered gut microbiota profiles [46,47,48]. Diet appears to be a key modulator of microbiota composition [50], making it a plausible therapeutic target in ADHD. The symptom improvements observed in our study may thus be mediated, at least in part, through diet-induced changes in immune or microbial pathways.

Additional support for a neurobiological mechanism comes from the BRAIN study by Hontelez et al. [51], which found increased activation in the precuneus—a brain region associated with attention regulation—following a few-foods diet in children with ADHD. Although no specific neural structures were isolated, the findings suggest that dietary interventions may alter brain function in relevant cortical networks.

### Limitations and Future Research

Despite the significant results demonstrating the impact of OD interventions on ADHD symptoms in children, several limitations of this study must be addressed.

Most critically, the absence of a control group limits our ability to definitively attribute the observed improvements solely to the oligoantigenic diet. Without including a comparator group that receives a placebo diet or another active intervention, it is difficult to exclude the potential impact of confounding variables or natural developmental changes. Although our study includes an internal categorization of participants into responders and non-responders based on a 40% reduction in ARS scores, it should be emphasized that this classification does not substitute for an external, randomized control group. Future investigations should employ a rigorously designed control condition to enable direct comparisons of outcomes. Such an approach would provide more definitive evidence regarding the efficacy of the diet and substantially strengthen the scientific foundation of dietary intervention research in ADHD.

Although the observed long-term symptom reductions following the four-week diet are clinically noteworthy, the limited sample size—particularly at follow-up—requires a cautious interpretation of the results. It is possible that the observed effects are partly influenced by sampling bias or other unmeasured variables. The number of participants decreased from 34 participants at baseline to 21 at follow-up, a common trend in long-term studies. Although dropout reasons were not systematically recorded, factors such as relocations, scheduling challenges, loss of interest, or changes in family circumstances may have contributed. It should be noted, however, that due to the small sample size and significant neurobiological maturation occurring around the age of 10, part of the observed improvements may be attributable to natural developmental processes. In addition, there was no a priori sample size calculation conducted for the repeated-measures ANOVA (rmANOVA), which limits the statistical conclusiveness of our findings. The small sample size increases the risk of both Type I and Type II errors and restricts the generalizability of the results. Future studies should therefore include formal power analyses and recruit larger samples to ensure sufficient statistical power for longitudinal analyses. Furthermore, ADHD symptoms were assessed exclusively via parent-report questionnaires, which may be subject to bias due to parental expectations or social desirability. Neither participants nor raters were blinded to the intervention, increasing the risk of expectancy effects. Lastly, the study sample consisted of self-selected families who voluntarily enrolled in a dietary intervention study, which may limit the generalizability of the findings to the broader ADHD population.

A number of reviews have discussed the OD as a treatment option in children with ADHD. Although the same studies were analyzed, the authors of several reviews have evaluated the effectiveness of the OD differently. Some authors described an unclear effectiveness of the OD, whereas others assessed the effectiveness to be relevant. These inconsistent evaluations emphasize the need of further studies on this subject [17,22,47].

Finally, it remains unclear which mechanisms underlie the observed effects of the oligoantigenic diet. Given the increasing interest in the gut–brain axis, future studies should explore how diet-induced changes—particularly in the gut microbiota—may affect neurodevelopment and behavior in ADHD. As suggested by Ly et al. [47], mechanistic research combining microbiome analyses, neuroimaging, and developmental assessments will be essential to fully understand the therapeutic potential of the oligoantigenic diet.

## 5. Conclusions

In summary, our study suggests that the identification and persistent avoidance of intolerant foods following the four-weeks of oligoantigenic diet may lead to lasting reductions in ADHD symptoms, particularly impulsivity, with effects persisting for up to 3.5 years. While the findings align with the previous research and highlight the potential of dietary interventions, the absence of a control group and small sample size limit the generalizability. Future randomized studies are needed to confirm these results and clarify underlying mechanisms.

## Figures and Tables

**Table 1:** Characteristics of participants at the end of the OD intervention and at the follow-up approximately 3.5 years later.

	Post-Diet	Follow-Up
**Included (*n*)**	**31**	**21**
Age (*M ± SD*) (Range)	10.50 ± 2.28 (7–15)	14.10 ± 2.09 (12–18)
Gender (m/f)	22/9	15/6
Comorbidities (ICD-10)	- Dyslexia (*F*81.0, *n* = 7) - Dyscalculia (*F*81.2, *n* = 2) - Oppositional Defiant Disorder (*F*91.3, *F*91.8, *n* = 2) - Autism (*F*84.0, *n* = 2) diagnosed in the course of this study - Encopresis (*F*98.1, *n* = 1) - Psoriasis (L40, *n* = 1) - Somnambulism (*F*51.3, *n* = 1) - Depersonalization and derealization syndrome (*F*48.1, *n* = 1) - Asperger Syndrome (*F*84.5, *n* = 1)	- Dyslexia (*F*81.0, *n* = 5) - Oppositional Defiant Disorder (*F*90, *n* = 1) - Enuresis (*F*98, *n* = 2) - Expressive language disorder (*F*80.1, *n* = 1) - Adjustment disorder (*F*43.2, *n* = 1) - Psoriasis (L40, *n* = 1) - Somnambulism (*F*51.3, *n* = 1) - Depersonalization and derealization syndrome (*F*48.1, *n* = 1) - Asperger Syndrome (*F*84.5, *n* = 1)

**Table 2:** Overview of the number of participants at various time points in this study.

Time Point	Number of Participants	Percentage
Pre-Diet	34	100%
Post-Diet	31	91%
Follow-Up	21	62%

**Table 3:** Medication status of responders and non-responders at follow-up.

Medication	Responders *n* = 14 (66%)	Non-Responders *n* = 7 (33%)
Yes	2 (14%)	3 (42%)
No	10 (72%)	2 (29%)
No Answer	2 (14%)	2 (29%)

Note. *n* = 21 participants.

**Table 4:** Statistics for the DISYPS-II FBB-ADHD (post-diet analysis).

	Time Points						
	Pre-Diet		Post-Diet		rmANOVA		
Scale	*M*	*SD*	*M*	*SD*	*F* (1. 30)	*p*	*d*
Total Score	1.72	0.52	1.03	0.62	51.37	0.000	1.20
Impulsivity	1.59	0.80	0.93	0.90	20.78	0.000	0.78
Inattention	2.06	0.54	1.32	0.60	54.86	0.000	1.32
Hyperactivity	1.36	0.75	0.73	0.69	28.81	0.000	0.87
Competence	0.69	0.47	0.94	0.59	11.02	0.001	−0.46

Note. *n* = 31 participants; *d* = Cohen’s *d*.

**Table 5:** Statistics for the DISYPS-II FBB-ADHD (follow-up analysis), *n* = 21.

	Time Points								
	Pre-Diet		Post-Diet		Follow-Up		rmANOVA		
Scale	*M*	*SD*	*M*	*SD*	*M*	*SD*	*F* (2. 40)	*p*	*η* ^2^ *_p_*
Total Score	1.69	0.52	0.86	0.49	0.81	0.45	39.64	0.000	0.42
Impulsivity	1.76	0.77	0.87	0.83	0.64	0.57	29.90	0.000	0.31
Inattention	2.03	0.50	1.13	0.49	1.16	0.43	34.72	0.000	0.45
Hyperactivity	1.22	0.76	0.51	0.51	0.45	0.60	20.06	0.000	0.24
Competence	0.66	0.49	0.99	0.57	1.05	0.62	6.23	0.004	0.09

**Table 6:** The “omnibus” test of the three symptom categories “inattention”, “hyperactivity”, and “impulsivity”, *n* = 21.

		Multivariate	Univariate
Interaction:	Symptom category × Time	*F*(4, 17) = 1.24, *p* = 0.332	*F*(4, 80) = 2.20, *p* = 0.076
Main effect:	Symptom category	*F*(2, 19) = 20.60, *p* < 0.001	*F*(2, 40) = 23.00, *p* < 0.001
Main effect:	Time	*F*(2, 19) = 28.71, *p* < 0.001	*F*(2, 40) = 40.63, *p* < 0.001

**Table 7:** DISYPS-II FBB-ADHD reduction depending on responder and medication (*n* = 21).

		Total Score					
		Pre-Diet		Post-Diet		Follow-Up	
Sample	*n*	*M*	*SD*	*M*	*SD*	*M*	*SD*
Responder	14	1.72	0.55	0.73	0.42	0.65	0.27
Non-Responder	7	1.64	0.48	1.14	0.52	1.12	0.58

## Data Availability

The original contributions presented in this study are included in the article. Further inquiries can be directed to the corresponding author.
